# Ozonation Eliminates Viable but Not Culturable *Legionella pneumophila* More Effectively than Chlorination

**DOI:** 10.3390/pathogens15090943

**Published:** 2026-09-05

**Authors:** Elisenda Arqué, Marina Simon-Coma, Pol Oliveras, Esteban Alberto Reynaga, Nieves Sopena, Maria Lluïsa Pedro-Botet, Noemí Párraga-Niño

**Affiliations:** 1 Clinical and Environmental Infectious Diseases Study Group, Germans Trias i Pujol Research Institute, Carretera de Can Ruti, Camí de les Escoles s/n, 08916 Badalona, Spain; earque@igtp.cat (E.A.); msimon@igtp.cat (M.S.-C.); pololiverasjulia@gmail.com (P.O.); eareynaga.germanstrias@gencat.cat (E.A.R.); nsopena.germanstrias@gencat.cat (N.S.); mlpbotet.germanstrias@gencat.cat (M.L.P.-B.); 2 Department of Infectious Diseases, Hospital Universitari Germans Trias i Pujol, Carretera de Can Ruti, 08916 Badalona, Spain; 3 Fundació Lluita Contra les Infeccions, Infectious Diseases Department, Hospital Universitari Germans Trias i Pujol, 08916 Badalona, Spain; 4 Department of Medicine, Universitat Autònoma de Barcelona, Carretera de Can Ruti, 08916 Badalona, Spain

**Keywords:** *Legionella pneumophila*, ozonation, chlorination, disinfection alternatives, water treatments

## Abstract

*Legionella pneumophila*, the causative agent of Legionnaires’ disease, remains a major public health concern in water systems. Chlorination is the most widely used disinfection strategy but presents important limitations, including reduced stability in hot water systems and the generation of carcinogenic by-products. This study evaluated ozonation as an alternative disinfection method, comparing its efficacy with chlorination against different physiological states of *L. pneumophila*, including culturable and viable but non-culturable (VBNC) cells, as well as biofilm-associated bacteria and amoebal hosts. The biocidal effect of ozone and chlorination on planktonic *L. pneumophila* was assessed by culture and viability-qPCR. Biofilm viability was evaluated using fluorescein diacetate staining, whereas amoeba viability was determined by flow cytometry. Ozonation achieved complete inactivation of both culturable and VBNC *L. pneumophila* within 5 min. In contrast, chlorination achieved complete loss of culturability only at high concentrations and was associated with the persistence of VBNC cells. Neither ozonation nor chlorination significantly reduced established biofilms or amoebae. These findings indicate that ozonation is a promising strategy for rapid inactivation of planktonic *L. pneumophila* and may overcome important limitations associated with chlorine-based treatments.

## 1. Introduction

Waterborne pathogens and their associated diseases are a major public health concern worldwide. *Legionella* is a Gram-negative bacterium ubiquitous in freshwater habitats, including lakes, streams, ponds, rivers and soil environments [1,2]. From natural environments, *Legionella* can colonize man-made water systems, where it can grow uncontrollably and be transmitted to humans by inhalation or microaspiration [3] of aerosols generated by showers, faucets, cooling towers, whirlpool spas and fountains. *Legionella* causes Legionnaires’ disease (LD), which is manifested as pneumonia or a mild nonpneumonic febrile illness called Pontiac fever [4]. Legionnaires’ disease is a notifiable disease in Spain and is subject to mandatory surveillance through the European Union and European Economic Area. The incidence of LD has increased by 135% in a 10-year period, with 14,056 confirmed cases in the European Union in 2024 and a case fatality rate exceeding 9% [5], highlighting its growing public health relevance.

The control of *Legionella* contamination is particularly important in healthcare settings, where immunocompromised individuals are at increased risk of infection and poor clinical outcomes [6]. Consequently, national and international consensus guidelines recommend the use of preventive measures to limit *Legionella* colonization of water systems, including thermal and chemical disinfection strategies such as chlorination, monochloramine or UV treatment [7]. While these approaches are effective in reducing planktonic bacterial populations, they often fail to achieve complete and sustained eradication of the microorganism [8,9].

A critical limitation of conventional disinfection methods is their ability to induce stress responses in *Legionella pneumophila*, including the transition to a viable but not culturable (VBNC) state [10]. In this state, cells remain metabolically active but are not detectable using standard culture-based methods, requiring alternative approaches such as molecular techniques or flow cytometry-based techniques combined with viability markers to distinguish viable from non-viable cells [11]. The VBNC state can be triggered by environmental stressors, including disinfectants such as chlorine [12], and has been associated with the persistence of *Legionella* in water systems. Importantly, VBNC cells can regain infectivity under favorable conditions, representing a hidden risk for public health [13]. Thus, assessing both culturability and viability is essential for an accurate evaluation of disinfection efficacy.

In addition to physiological adaptations such as the VBNC state, *Legionella* persistence is further enhanced by its association with environmental reservoirs, including biofilms [14] and intracellular replication within amoebae [15,16], which confer protection against environmental stress [17]. Current disinfection strategies are generally more effective against planktonic cells than against these protected forms, contributing to recurrent contamination even after treatment [8,18].

At present, the most effective method to perform shock disinfection in hot water systems is thermal shock. Thermal shock has been shown to reduce colonization by *Legionella* more effectively than chlorination, monochloramine or hydrogen peroxide [19]. However, none of these methods are effective unless followed up by a proper maintenance regime.

Ozonation has been proposed as a promising alternative to chlorination for water disinfection. Its main advantage is that it does not generate carcinogenic, mutagenic and/or toxic decomposition by-products [20] and it does not corrode any pipe material. Another important feature is ozone’s stability and effectiveness across a range of temperatures [21], including conditions in which chlorine rapidly dissipates. Ozone is a strong oxidizing agent and has proved antimicrobial activity; however, its effectiveness against *Legionella* appears to depend strongly on the concentration and exposure conditions applied. Previous studies have reported reductions in bacterial load following ozonation [21,22,23], but these studies have often involved long exposure times or incompletely characterized treatment conditions, limiting their applicability.

In particular, there remains a lack of knowledge regarding the effectiveness of short-term ozonation treatments and their impact on different physiological states of *Legionella*, including VBNC cells. Given the potential role of these cells in infection risk and the limitations associated with conventional disinfection strategies, the objective of this study was to evaluate the efficacy of ozonation compared to chlorination in *L. pneumophila* suspensions by assessing post-treatment culturability and viability, with particular attention to VBNC planktonic *Legionella*. Additionally, the impact of these treatments on biofilm and amoebal hosts was assessed to provide a broader understanding of their role in *Legionella* persistence.

## 2. Materials and Methods

### 2.1. Bacterial Samples and Cultivation

An environmental *L. pneumophila* sg 1 isolate was used. It was grown on buffered charcoal yeast extract culture plates (BCYE, Oxoid; Thermo Fisher Scientific, Waltham, MA, USA) for 72 h at 37 °C.

### 2.2. Amoebal Strain and Culture Conditions

*Acanthamoeba castellanii* (ATCC 30234) was grown axenically in PYG medium [24] supplemented with penicillin–streptomycin (50–100 U/mL) to prevent bacterial contamination. Cultures were maintained at 30 °C in 5% CO_2_ for 2–3 days until confluent growth of trophozoites was observed. For encystation, 5 × 10^5^ trophozoites were seeded in 24-well plates in encystation medium [25,26] and incubated for 10 days at 25 °C in 5% CO_2_ until cyst formation was confirmed.

### 2.3. Biocide Treatments

Ozonation was compared with chlorination using hypochlorite as the reference disinfection method. Free chlorine concentration was measured using a HI701 Free Chlorine Checker kit (Hanna Instruments, Woonsocket, RI, USA) according to the manufacturer’s instructions. Two chlorine concentrations were tested: standard chlorination (0.6–0.8 ppm) and hyperchlorination (2 ppm). Hypochlorite was added once at the beginning of each experiment in sterile distilled water. Ozone treatment was performed using an O.zone^®^ (Sabadell, Spain) air-fed ozonator with a nominal production of 0.5 g/h. Ozonised water was generated and used to prepare the bacterial and amoebal suspensions. Redox potential was monitored during each experiment. Biocidal activity was neutralized by the addition of sodium thiosulfate (24 mg/L final concentration) in accordance with UNE 100030 [27].

### 2.4. Legionella pneumophila Planktonic Assay

The biocidal effect of ozone and chlorine was first evaluated on planktonic *Legionella pneumophila*. A bacterial suspension of 10^4^ colony-forming units (CFU)/mL was treated with ozonised water or chlorine at different concentrations (0.6–0.8 ppm and 2 ppm) and incubation times (5 and 15 min, and 1 and 24 h) at 37 °C with agitation (150 rpm). Culturability and viability were subsequently assessed.

This concentration was selected to challenge the disinfectant treatments under a high bacterial load, corresponding to approximately 10^7^ CFU/L, which is close to the upper limit of direct quantification routinely achieved by culture-based methods according to ISO 11731 [28].

#### 2.4.1. Effect on Culturability

Culturability was assessed by plate count. Bacterial suspensions were serially diluted in sterile water and plated onto BCYE agar. Plates were incubated at 37 °C for 72 h, and CFUs were determined.

#### 2.4.2. Effect on Viability

Bacterial viability was assessed using PMA-qPCR in those conditions where no culturable cells were detected. In order to inhibit amplification of DNA from dead cells, samples were treated with 50 μM propidium monoazide (PMA; Biotium, Fremont, CA, USA) and incubated in the dark for 15 min, followed by photoactivation using a PhastBlue device (Geniul, Barcelona, Spain). DNA was extracted using a Chelex-based protocol. In parallel, a non-PMA-treated sample for each condition was used as a control for total DNA. Quantitative PCR targeting the *Legionella* 16S rRNA (Forward: 5′-GACGATCGGTAGCTGGTCTG-3′; Reverse: 5′-CTCCTCCCCACTGAAAGTGC-3′) gene was performed using a LightCycler 480 System and LightCycler 480^®^ Software version 1.5.1.62 SP3 (Roche Diagnostics GmbH, Basel, Switzerland) with SYBR Green detection of treated and non-treated samples. Quantification was based on a standard curve generated from seven tenfold serial dilutions of *L. pneumophila* ranging from 10^1^ to 10^7^ cells/mL. The initial concentration was determined by plate counts from a freshly prepared exponential-phase culture, and the serial dilutions were validated by culture. Molecular-grade water was included as a no-template control in all qPCR runs. The standard curve showed a coefficient of determination (*R^2^*) of 0.9969 and a qPCR efficiency of 109%.

#### 2.4.3. Data Analysis

The qPCR signal was considered to represent total cell counts, whereas PMA-qPCR quantified viable cells. The difference between viable (PMA-qPCR) and culturable (plate count) cells was interpreted as the proportion of viable but non-culturable (VBNC) bacteria. Differences between total and viable cells were attributed to dead cells. Biocidal activity was expressed as the reduction in viable cells relative to untreated controls. All experiments were performed in triplicate.

### 2.5. Biocide Assay in Legionella pneumophila Biofilm

#### 2.5.1. Biofilm Formation and Treatment

*L. pneumophila* biofilms were generated in 96-well plates (NUNC Microwell 96F, Thermo Fisher Scientific, Waltham, MA, USA) by incubating 150 μL of bacterial suspension (10^7^ cells/mL) in BYE medium at 30 °C for 96 h without agitation. Biofilm formation was confirmed after 96 h by fluorescein diacetate (FDA) staining, which demonstrated the presence of a metabolically active surface-associated bacterial population. The 96 h incubation period was therefore selected as the endpoint for biofilm establishment prior to biocide treatment.

Following biofilm formation, biocide treatments were applied for 24 h at different concentrations (0.1, 0.2, 1 and 2 ppm for chlorine; 600, 700, 800, 900 and 1000 mV for ozone). Control wells without biocide and blank wells without bacterial suspension were included.

#### 2.5.2. Biofilm Quantification

Biofilm viability was assessed using fluorescein diacetate (FDA) staining. After treatment, wells were washed with PBS and incubated with FDA (20 μg/mL) for 30 min at 37 °C in the dark. Fluorescence was measured using a Varioskan^TM^ microplate reader (Thermo Fisher Scientific, Waltham, MA, USA) (λex 494 nm, λem 518 nm). Biocidal activity was expressed as the percentage reduction in biofilm relative to untreated controls. All experiments were performed in triplicate.

### 2.6. Biocide Assay in Acanthamoeba castellanii

#### 2.6.1. Trophozoites

*A. castellanii* trophozoites were cultured in PYG medium and seeded in 24-well plates (5 × 10^5^ cells/well) [24]. After 2 h of attachment at 30 °C, the medium was replaced with biocide solutions at different concentrations and incubated for 24 h at 30 °C. Untreated amoebae were used as controls. Cell viability was assessed by propidium iodide (PI) staining followed by flow cytometry using a FACSCanto II flow cytometer and BD FACSDiva^TM^ software (BD Biosciences, Erembodegem, Belgium). Biocidal activity was expressed as the percentage of trophozoite mortality relative to untreated controls.

#### 2.6.2. Cysts

Cysts were obtained by encystation in encystation medium and confirmed by microscopy and flow cytometry. Cyst suspensions (10^4^ cells/mL) were seeded in 24-well plates and treated with biocides for 24 h at 25 °C. Untreated samples were used as controls. Cell viability was assessed by PI staining and analyzed by flow cytometry using a FACSCanto II flow cytometer and BD FACSDiva^TM^ software (BD Biosciences, Erembodegem, Belgium). Biocidal activity was expressed as the percentage of cyst mortality relative to untreated controls. All experiments were performed in triplicate.

## 3. Results

### 3.1. Ozonation Rapidly Inactivates Planktonic Legionella pneumophila

The biocidal activity of ozonation against planktonic *L. pneumophila* was first evaluated using culture-based methods. At a redox potential of 750 mV, ozonation resulted in the complete loss of culturability of *L. pneumophila* after 5 min of exposure. Chlorine concentrations of 0.6–0.8 ppm did not achieve this bactericidal effect even after 24 h. However, hyperchlorination (2 ppm) achieved a 100% reduction in culturable cells at this timepoint (Table 1).

### 3.2. Ozonation Eliminates Viable but Not-Culturable (VBNC) Legionella pneumophila Cells, Unlike Chlorination

**Figure 1:**
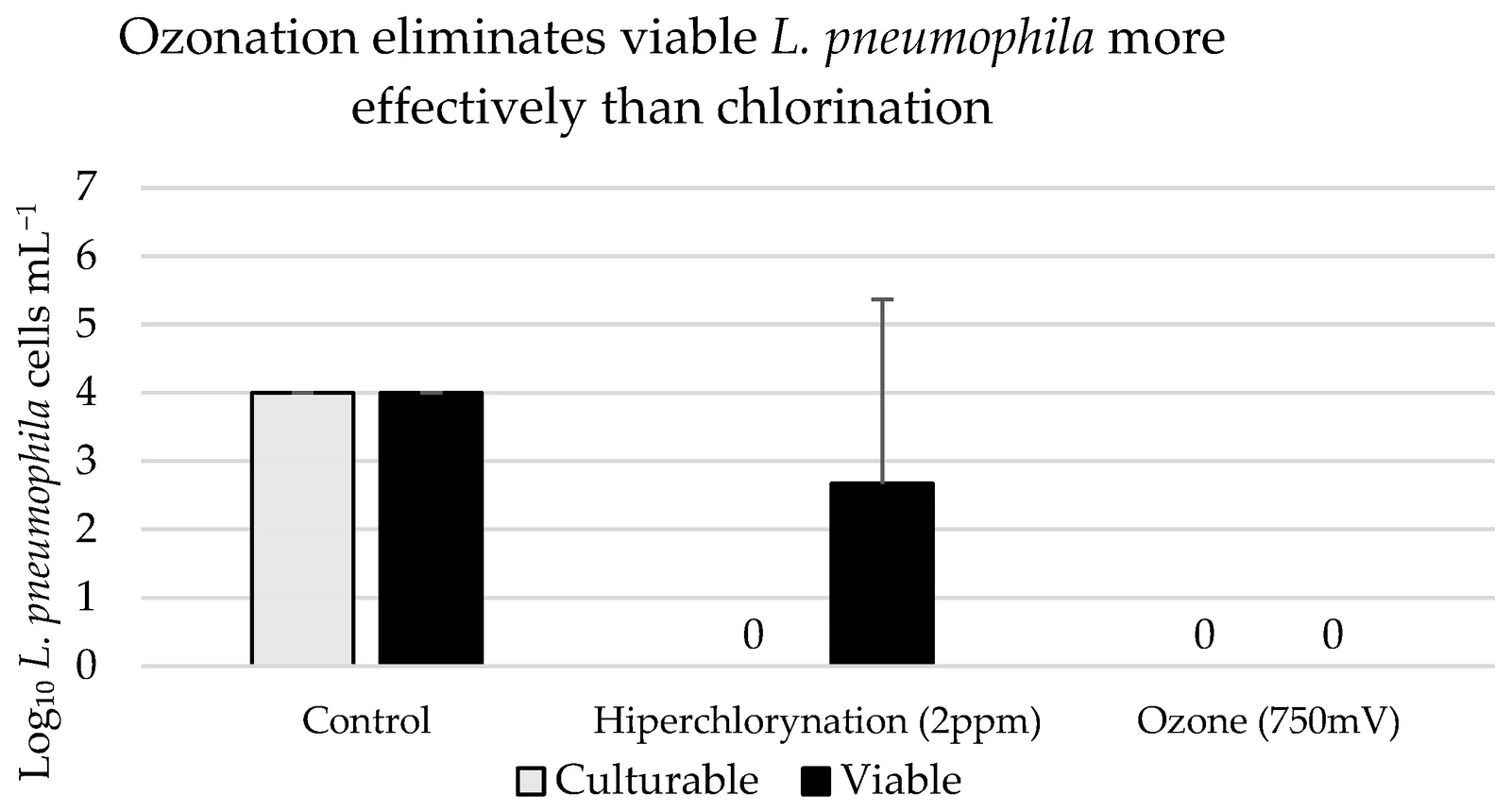
Culturable and viable *Legionella pneumophila* (log_10_ cells mL^−1^) after 5 min of hyperchlorination (2 ppm) or ozonation (750 mV). Error bars indicate standard deviation.

The effects of ozonation and hyperchlorination on *L. pneumophila* viability were further assessed using viability qPCR. Ozone treatment resulted in the complete loss of detectable viable cells after 5 min of exposure. In contrast, chlorine (2 ppm) achieved only a reduction of 1 logarithm at the same time point (Figure 1) and required 15 min to reach an inactivation level comparable to that obtained with ozone after 5 min.

Notably, the discrepancy between plate counts and viability qPCR measurements following chlorine treatment indicated the presence of VBNC cells. While hyperchlorination eliminated culturability, viable cells remained detectable by PMA qPCR, suggesting induction of the VBNC state. In contrast, ozone treatment did not result in detectable VBNC cells under the conditions tested, indicating a more complete inactivation of metabolically active bacteria.

### 3.3. Resistant Forms Show Tolerance to Both Treatments

#### 3.3.1. Biofilm-Associated *Legionella pneumophila*

**Figure 2:**
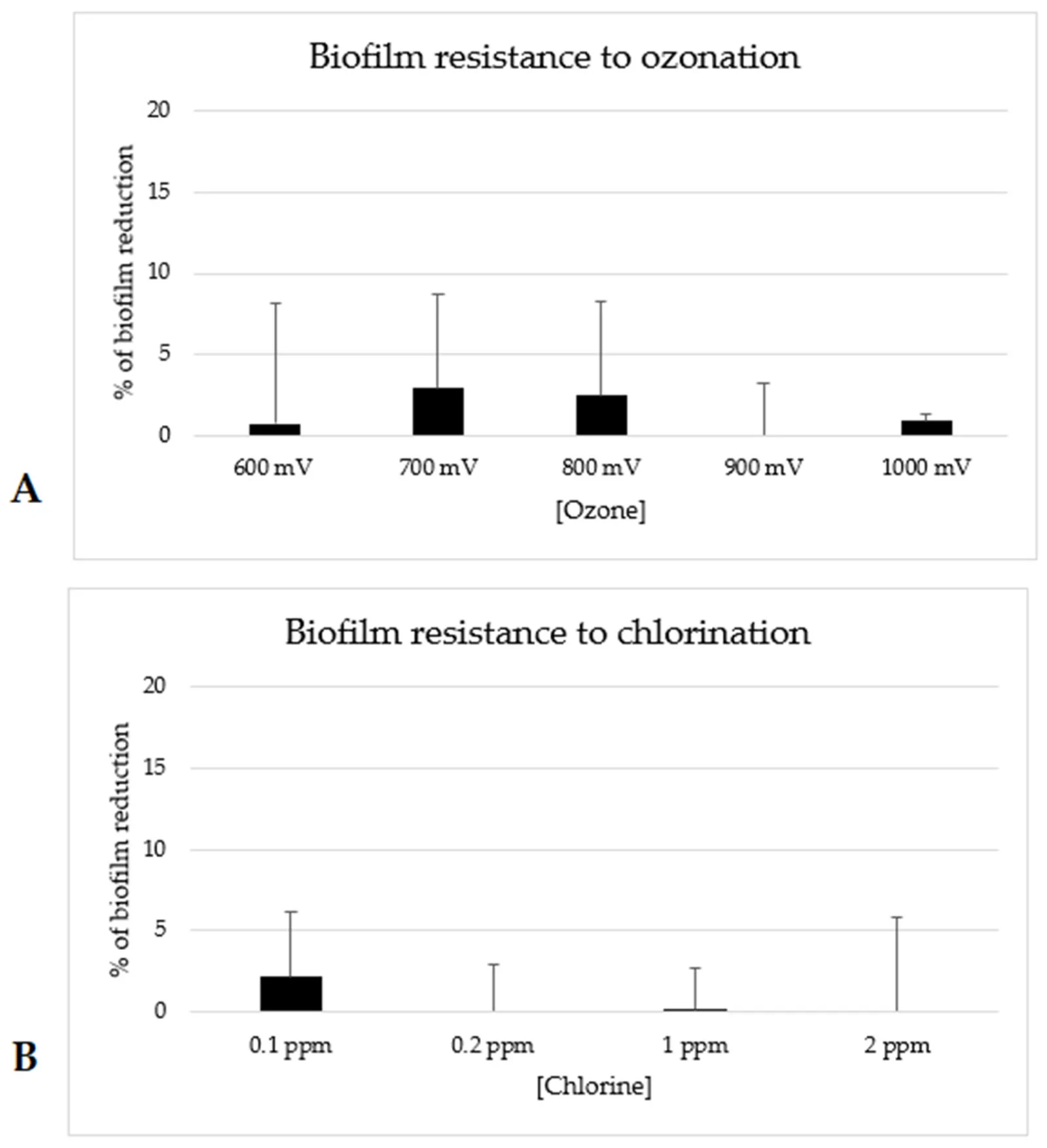
Reduction (%) of *Legionella pneumophila* biofilm following treatment with ozone (mV) (**A**) and chlorine (ppm) (**B**) relative to untreated controls.

The effect of ozonation and chlorination on *L. pneumophila* biofilms was evaluated across a range of concentrations (0.1, 0.2, 1 and 2 ppm for chlorine; 600, 700, 800, 900 and 1000 mV for ozone). Neither treatment resulted in a significant reduction in biofilm-associated cells, even at increased ozone redox potentials or chlorine concentrations (Figure 2). These findings are consistent with the well-documented resistance of biofilm structures to chemical disinfection, highlighting their role as persistent environmental reservoirs [24,25].

#### 3.3.2. *Acanthamoeba castellanii*

The susceptibility of *A. castellanii*, a known host of *Legionella* [15,16], was assessed in both trophozoite and cyst forms. Neither ozonation nor chlorine treatment resulted in substantial mortality in either life stage (Table 2). The resistance observed in both trophozoites and cysts supports their role as protective reservoirs that may contribute to *Legionella* persistence in water systems.

## 4. Discussion

Waterborne pathogens such as *Legionella pneumophila* remain a significant public health concern, particularly in engineered water systems where conditions favor their proliferation and persistence. In this study, we compared the effectiveness of ozone and chlorine treatments under controlled in vitro conditions, with a specific focus on bacterial viability and the induction of VBNC states.

Ozonation (750 mV) demonstrated a rapid biocidal action against planktonic *L. pneumophila*, achieving complete inactivation of culturable bacteria within 5 min. In contrast, standard chlorination conditions (0.6–0.8 ppm), commonly used for continuous disinfection, were insufficient, resulting in the persistence of culturable bacteria even after prolonged exposure times (1 and 24 h). This finding highlights limitations in current disinfection strategies and associated public health concerns. Conversely, hyperchlorination (2 ppm), typically applied as a shock treatment, was effective in eliminating culturable cells within 5 min.

In addition to this effect on culturability, a key finding of this study is the differential effect of ozone and chlorine on *L. pneumophila* viability assessed using PMA-qPCR. Chlorination, even at higher concentrations, resulted in the persistence of viable cells despite the absence of detectable growth in culture and it required longer exposure to achieve a complete reduction in viability, indicating the transition to the VBNC state. In contrast, ozonation led to the complete loss of detectable viable cells in 5 min of exposure, suggesting a more effective inactivation of planktonic bacteria.

The induction of the VBNC state following chlorination is particularly relevant from a public health perspective. VBNC *Legionella* cells remain metabolically active and may regain infectivity under favorable conditions [29], thereby representing a hidden reservoir that is not detected by conventional culture-based monitoring methods. Our results support previous observations indicating that disinfection strategies based solely on culturability may overestimate treatment efficacy [12,13]. By combining plate counts and viability-qPCR, this study provides a more comprehensive evaluation of biocidal activity and highlights the importance of considering VBNC cells in *Legionella* control strategies.

Consistent with our findings, a five-year investigation of hyperchlorination in a *Legionella*-colonized plumbing system reported persistent positive samples following treatment [30], indicating that this approach may not achieve complete eradication of the pathogen, potentially due to the VBNC state induction and subsequent recolonization of the water system. Similarly, although thermal shock is considered one of the most effective methods for shock disinfection of hot water systems, its success depends on an adequate maintenance regime and its implementation may be limited by facility characteristics such as the number of floors or the water heating system. Furthermore, previous studies have shown that some *Legionella* strains can survive thermal shock treatments, possibly through the induction of the VBNC state [31,32].

Our findings are in line with previous studies reporting the antimicrobial potential of ozone, although many have focused exclusively on culturability. For example, Domingue et al. reported that ozone achieved a 99% reduction in *L. pneumophila* within 5 min, whereas chlorine required longer exposure times, likely due to differences in concentration [33]. However, the absence of viability assessment in previous studies limits the interpretation of their results. Similarly, studies evaluating ozonation in water systems have reported reductions in *Legionella* concentrations [18], but without assessing VBNC cells, which may contribute to recolonization events observed after treatment.

Despite its effectiveness against planktonic cells, ozonation did not significantly reduce biofilm-associated *L. pneumophila* or *A. castellanii* in either trophozoite or cyst forms. This observation is consistent with the well-established resistance of biofilms and protozoan hosts to chemical disinfection, which act as protective reservoirs for *Legionella* [34,35]. These results highlight that neither ozonation nor chlorination alone is sufficient to eliminate all bacterial reservoirs at the exposure times tested. However, prolonged or continuous treatments might have an effect on established reservoirs.

From an applied perspective, ozonation presents several advantages over chlorination. Unlike chlorine, ozone does not generate chlorinated disinfection by-products and maintains its efficacy at elevated temperatures [20], making it particularly suitable for hot water systems. In addition, its ability to eliminate VBNC cells suggests that it could reduce the risk of undetected bacterial persistence and subsequent recolonization. However, ozonation systems typically require a higher initial investment, and their implementation should be carefully optimized depending on system characteristics and water quality. Although ozonation showed superior efficacy against planktonic *L. pneumophila* under the experimental conditions tested, its implementation in real water systems requires consideration of water chemistry. In particular, ozone may promote bromate formation in waters containing bromide ions [36]. Consequently, the long-term control of *Legionella* may require combined or complementary treatment strategies. Furthermore, previous studies have reported that ozonation followed by chlorination can influence the formation of disinfection by-products [37]. These aspects were not evaluated in the present study and should be considered in future field-scale investigations.

The results obtained in this study suggest that ozonation reduces the presence of planktonic *Legionella*, but it could also reduce the presence of other microorganisms because it targets bacterial membranes. Ozone would therefore be a good candidate in the fight against multiresistant microorganisms found in water such as *Pseudomonas* [33], *Klebsiella*, *Serratia*, *Acinetobacter* and *Mycobacterium chelonae* [38].

This study has some limitations that should be considered. First, experiments were conducted under in vitro conditions that do not fully replicate the complexity of real water distribution systems, including the presence of organic matter and heterogeneous microbial communities. Second, biocidal activity was assessed during single treatment cycles without continuous replenishment of the disinfectant. Further studies using pilot-scale systems and real-world conditions are needed to confirm these findings and to determine optimal operational parameters.

## 5. Conclusions

This study demonstrates that while both chlorination and ozonation effectively eliminate culturable *L. pneumophila*, they differ substantially in their impact on bacterial viability. Ozonation achieved complete inactivation of both culturable and viable cells and did not promote the VBNC state, whereas chlorination was associated with the persistence of VBNC cells. These findings highlight the importance of incorporating viability-based methods in the evaluation of disinfection strategies and support the potential of ozonation as a promising alternative for water disinfection by reducing *Legionella* presence in terminal points.

## Figures and Tables

**Table 1:** Reduction in culturability of a *L. pneumophila* suspension (10^4^ CFU/mL) by ozonised water and chlorine treatments at different exposure times. Results are expressed as the % of bacterial reduction compared with a control without biocide treatment. Standard deviation is expressed as ±. Each experiment was performed in triplicate.

Biocide	Concentration	5 min	15 min	1 h	24 h
Chlorine	0.6–0.8 ppm	18 ± 27	25 ± 28	42 ± 11	89 ± 2
	2 ppm	100	100	100	100
Ozone	750 mV	100	100	100	100

**Table 2:** Mortality (%) of *Acanthamoeba castellanii* trophozoites and cysts following ozonation and chlorination relative to untreated controls (mean ± SD, *n* = 3).

Reservoir	Ozone (750 mV)	Chlorine (2 ppm)
Trophozoites	1.24 ± 0.37	0.13 ± 0.81
Cysts	2.47 ± 2.45	3.17 ± 2.64

## Data Availability

The original contributions presented in this study are included in the article. Further inquiries can be directed to the corresponding author.

## Abbreviations

The following abbreviations are used in this manuscript:

VBNC	Viable but non-culturable
LD	Legionnaire’s disease
CFU	Colony-forming units
FDA	Fluorescein diacetate
PI	Propidium iodide
